# The Measurement of Emotional Intelligence: A Critical Review of the Literature and Recommendations for Researchers and Practitioners

**DOI:** 10.3389/fpsyg.2019.01116

**Published:** 2019-05-28

**Authors:** Peter J. O'Connor, Andrew Hill, Maria Kaya, Brett Martin

**Affiliations:** ^1^School of Management, QUT Business School, Queensland University of Technology, Brisbane, QLD, Australia; ^2^Clinical Skills Development Service, Metro North Hospital and Health Service, Queensland Health, Brisbane, QLD, Australia; ^3^School of Psychology, Faculty of Health and Behavioural Sciences, The University of Queensland, St. Lucia, QLD, Australia; ^4^School of Advertising, Marketing and Public Relations, QUT Business School, Queensland University of Technology, Brisbane, QLD, Australia

**Keywords:** emotional intelligence, measures, questionnaires, trait, ability, mixed, recommendations

## Abstract

Emotional Intelligence (EI) emerged in the 1990s as an ability based construct analogous to general Intelligence. However, over the past 3 decades two further, conceptually distinct forms of EI have emerged (often termed “trait EI” and “mixed model EI”) along with a large number of psychometric tools designed to measure these forms. Currently more than 30 different widely-used measures of EI have been developed. Although there is some clarity within the EI field regarding the types of EI and their respective measures, those external to the field are faced with a seemingly complex EI literature, overlapping terminology, and multiple published measures. In this paper we seek to provide guidance to researchers and practitioners seeking to utilize EI in their work. We first provide an overview of the different conceptualizations of EI. We then provide a set of recommendations for practitioners and researchers regarding the most appropriate measures of EI for a range of different purposes. We provide guidance both on how to select and use different measures of EI. We conclude with a comprehensive review of the major measures of EI in terms of factor structure, reliability, and validity.

## Overview and Purpose

The purpose of this article is to review major, widely-used measures of Emotional Intelligence (EI) and make recommendations regarding their appropriate use. This article is written primarily for academics and practitioners who are not currently experts on EI but who are considering utilizing EI in their research and/or practice. For ease of reading therefore, we begin this article with an introduction to the different types of EI, followed by a brief summary of different measures of EI and their respective facets. We then provide a detailed set of recommendations for researchers and practitioners. Recommendations focus primarily on choosing between EI constructs (ability EI, trait EI, mixed models) as well as choosing between specific tests. We take into account such factors as test length, number of facets measured and whether tests are freely available. Consequently we also provide recommendations both for users willing to purchase tests and those preferring to utilize freely available measures.

In our detailed literature review, we focus on a set of widely used measures and summarize evidence for their validity, reliability, and conceptual basis. Our review includes studies that focus purely on psychometric properties of EI measures as well as studies conducted within applied settings, particularly health care settings. We include comprehensive tables summarizing key empirical studies on each measure, in terms of their research design and main findings. Our review includes measures that are academic and/or commercial as well as those that are freely available or require payment. To assist users with accessing measures, we include web links to complete EI questionaries for freely available measures and to websites and/or example items for copyrighted measures. For readers interested in reviews relating primarily to EI constructs, theory and outcomes rather than specifically *measures* of EI, we recommend a number of recent high quality publications (e.g., Kun and Demetrovics, 2010; Gutiérrez-Cobo et al., 2016). Additionally, for readers interested in a review of measures without the extensive recommendations we provide here, we recommend the chapter by Siegling et al. (2015).

## Early Research on Emotional Intelligence

EI emerged as a major psychological construct in the early 1990s, where it was conceptualized as a set of abilities largely analogous to general intelligence. Early influential work on EI was conducted by Salovey and Mayer (1990), who defined EI as the “the ability to monitor one's own and others' feelings and emotions, to discriminate among them and to use this information to guide one's thinking and actions” (p. 189). They argued that individuals high in EI had certain emotional abilities and skills related to appraising and regulating emotions in the self and others. Accordingly, it was argued that individuals high in EI could accurately perceive certain emotions in themselves and others (e.g., anger, sadness) and also regulate emotions in themselves and others in order to achieve a range of adaptive outcomes or emotional states (e.g., motivation, creative thinking).

However, despite having a clear definition and conceptual basis, early research on EI was characterized by the development of multiple measures (e.g., Bar-On, 1997a,b; Schutte et al., 1998; Mayer et al., 1999) with varying degrees of similarity (see Van Rooy et al., 2005). One cause of this proliferation was the commercial opportunities such tests offered to developers and the difficulties faced by researchers seeking to obtain copyrighted measures (see section Mixed EI for a summary of commercial measures). A further cause of this proliferation was the difficulty researchers faced in developing measures with good psychometric properties. A comprehensive discussion of this issue is beyond the scope of this article (see Petrides, 2011 for more details) however one clear challenge faced by early EI test developers was constructing emotion-focused questions that could be scored with objective criteria. In comparison to measures of cognitive ability that have objectively right/wrong answers (e.g., mathematical problems), items designed to measure emotional abilities often rely on expert judgment to define correct answers which is problematic for multiple reasons (Roberts et al., 2001; Maul, 2012).

A further characteristic of many early measures was their failure to discriminate between measures of typical and maximal performance. In particular, some test developers moved away from pure ability based questions and utilized self-report questions (i.e., questions asking participants to rate behavioral tendencies and/or abilities rather than objectively assessing their abilities; e.g., Schutte et al., 1998). Other measures utilized broader definitions of EI that included social effectiveness in addition to typical EI facets (see Ashkanasy and Daus, 2005) (e.g., Boyatzis et al., 2000; Boyatzis and Goleman, 2007). Over time it became clear that these different measures were tapping into related, yet distinct underlying constructs. Currently, there are two popular methods of classifying EI measures. First is the distinction between trait and ability EI proposed initially by Petrides and Furnham (2000) and further clarified by Pérez et al. (2005). Second is in terms of the three EI “streams” as proposed by Ashkanasy and Daus (2005). Fortunately there is overlap between these two methods of classification as we discuss below.

## Methods of Classifying EI

The distinction between *ability EI* and *trait EI first* proposed by Petrides and Furnham (2000) was based purely on whether the measure was a test of maximal performance (ability EI) or a self-report questionnaire (trait EI) (Petrides and Furnham, 2000; Pérez et al., 2005). According to this method of classification, Ability EI tests measure constructs related to an individual's *theoretical* understanding of emotions and emotional functioning, whereas trait EI questionnaires measure typical behaviors in emotion-relevant situations (e.g., when an individual is confronted with stress or an upset friend) as well as self-rated abilities. Importantly, the key aspect of this method of classification is that EI type is best defined by method of measurement: all EI measures that are based on self-report items are termed “trait EI” whereas all measures that are based on maximal performance items are termed “ability EI”.

The second popular method of classifying EI measures refers the three EI “streams” (Ashkanasy and Daus, 2005). According to this method of classification, stream 1 includes ability measures based on Mayer and Salovey's model; stream 2 includes self-report measures based on Mayer and Salovey's model and stream 3 includes “expanded models of emotional intelligence that encompass components not included in Salovey and Mayer's definition” (p. 443). Ashkanasy and Daus (2005) noted that stream 3 had also been referred to as “mixed” models in that they comprise a mixture of personality and behavioral items. The term “mixed EI” is now frequently used in the literature to refer to EI measures that measure a combination of traits, social skills and competencies and overlaps with other personality measures (O'Boyle et al., 2011).

Prior to moving on, we note that Petrides and Furnham's (2000) trait vs. ability distinction is sufficient to categorize the vast majority of EI tests. Utilizing this system, both stream 2 (self-report) and stream 3 (self-report mixed) are simply classified as “trait” measures. Indeed as argued by Pérez et al. (2005), this method of classification is probably sufficient given that self-report measures of EI tend to correlate strongly regardless of whether they are stream 2 or stream 3 measures. However, given that the terms “stream 3” and “mixed” are so extensively used in the EI literature, we will also use them here. We are not proposing that these terms are ideal or even useful when classifying EI, but rather we wish to adopt language that is most representative of the existing literature on EI. In the following section therefore, we refer to ability EI (stream 1), trait EI (steam 2), and mixed EI (stream 3). As outlined later, decisions regarding which measure of EI to use should be based on what form of EI is relevant to a particular research project or professional application.

## Ability EI

For the purposes of this review, we refer to “ability” based measures as tests that utilize questions/items comparable to those found in IQ tests (see Austin, 2010). These include all tests containing ability-type items and not only those based directly on Mayer and Salovey's model. In contrast to trait based measures, ability measures do not require that participants self-report on various statements, but rather require that participants solve emotion-related problems that have answers that are deemed to be correct or incorrect (e.g., what emotion might someone feel prior to a job interview? (a) sadness, (b) excitement, (c) nervousness, (d) all of the above). Ability based measures give a good indication of individuals' ability to understand emotions and how they work. However since they are tests of maximal ability, they do not tend to predict typical behavior as well as trait based measures (see O'Connor et al., 2017). Nevertheless, ability-based measures are valid, albeit weak, predictors of a range of outcomes including work related attitudes such as job satisfaction (Miao et al., 2017), and job performance (O'Boyle et al., 2011).

## Trait EI

In this review, we define trait based measures as those that utilize self-report items to measure overall EI and its sub dimensions. We utilize this term for measures that are self-report, and have not explicitly been termed as “mixed” or “stream 3” by others. Individuals high in various measures of trait EI have been found to have high levels of self-efficacy regarding emotion-related behaviors and tend to be competent at managing and regulating emotions in themselves and others. Also, since trait EI measures tend to measure typical behavior rather than maximal performance, they tend to provide a good prediction of actual behaviors in a range of situations (Petrides and Furnham, 2000). Recent meta-analyses have linked trait EI to a range of work attitudes such as job satisfaction and organization commitment (Miao et al., 2017), Job Performance (O'Boyle et al., 2011).

## Mixed EI

As noted earlier, although the majority of EI measures can be categorized using the terms “ability EI” and “trait EI”, we adopt the term “mixed EI” in this review when this term has been explicitly used in our source articles. The term mixed EI is predominately used to refer to questionnaires that measure a combination of traits, social skills and competencies that overlap with other personality measures. Generally these measures are self-report, however a number also utilize 360 degree forms of assessment (self-report combined with multiple peer reports from supervisors, colleagues and subordinates) (e.g., Bar-On, 1997a,b) This is particularly true for commercial measures designed to predict and improve performance in the workplace. A common aspect in many of these measures is the focus on emotional “competencies” which can theoretically be developed in individuals to enhance their professional success (See Goleman, 1995). Research on mixed measures have found them to be valid predictors of multiple emotion-related outcomes including job satisfaction, organizational commitment (Miao et al., 2017), and job performance (O'Boyle et al., 2011). Effect sizes of these relationships tend to be moderate and on par with trait-based measures.

We note that although different forms of EI have emerged (trait, ability, mixed) there are nevertheless a number of conceptual similarities in the majority of measures. In particular, the majority of EI measures are regarded as *hierarchical* meaning that they produce a total “EI score” for test takers along with scores on multiple facets/subscales. Additionally, the facets in ability, trait and mixed measures of EI have numerous conceptual overlaps. This is largely due to the early influential work of Mayer and Salovey. In particular, the majority of measures include facets relating to (1) perceiving emotions (in self and others), (2) regulating emotions in self, (3) regulating emotions in others, and (4) strategically utilizing emotions. Where relevant therefore, this article will compare how well different measures of EI assess the various facets common to multiple EI measures.

## Emotional Intelligence Scales

The following emotional intelligence scales were selected to be reviewed in this article because they are all widely researched general measures of EI that also measure several of the major facets common to EI measures (perceiving emotions, regulating emotions, utilizing emotions).

1. Mayer-Salovey-Caruso Emotional Intelligence Tests (MSCEIT) (Mayer et al., 2002a,b).
2. Self-report Emotional Intelligence Test (SREIT) (Schutte et al., 1998)
3. Trait Emotional Intelligence Questionnaire (TEIQue) (Petrides and Furnham, 2001)
4. Bar-On Emotional Quotient Inventory (EQ-i) (Bar-On, 1997a,b)
5.
  - The Situational Test of Emotional Management (STEM) (MacCann and Roberts, 2008)
  - The Situational Test of Emotional Understanding (STEU) (MacCann and Roberts, 2008)
6. Emotional and Social competence Inventory (ESCI) (Boyatzis and Goleman, 2007)

The complete literature review of these measures is included in the Literature Review section of this article. The following section provides a set of recommendations regarding which of these measures is appropriate to use across various research and applied scenarios.

## Recommendations Regarding the Appropriate Use of Measures

### Deciding Between Measuring Trait EI, Ability EI and Mixed EI

A key decision researchers/practitioners need to make prior to incorporating EI measures into their work is whether they should utilize a trait, ability or mixed measure of EI. In general, we suggest that when researchers/practitioners are interested in emotional abilities and competencies then they should utilize measures of ability EI. In particular ability EI is important in situations where a good theoretical understanding of emotions is required. For example a manager high in ability EI is more likely to make good decisions regarding team composition. Indeed numerous studies on ability EI and decision making in professionals indicates that those high in EI tend to be competent decision makers, problem solvers and negotiators due primarily to their enhanced abilities at perceiving and understanding emotions (see Mayer et al., 2008). More generally, ability EI research also has demonstrated associations between ability EI and social competence in children (Schultz et al., 2004) and adults (Brackett et al., 2006).

We suggest that researchers/practitioners should select trait measures of EI when they are interested in measuring behavioral tendencies and/or emotional self-efficacy. This should be when ongoing, typical behavior is likely to lead to positive outcomes, rather than intermittent, maximal performance. For example, research on task-induced stress (i.e., temporary states of negative affect evoked by short term, challenging tasks) has shown trait EI to have incremental validity over other predictors (O'Connor et al., 2017). More generally, research tends to show that trait EI is a good predictor of effective coping styles in response to life stressors (e.g., Austin et al., 2010). Overall, trait EI is associated with a broad set of emotion and social related outcomes adults and children (Mavroveli and Sánchez-Ruiz, 2011; Petrides et al., 2016) Therefore in situations characterized by ongoing stressors such as educational contexts and employment, we suggest that trait measures be used.

When both abilities and traits are important, researchers/practitioners might choose to use *both* ability and trait measures. Indeed some research demonstrates that both forms of EI are important stress buffers and that they exert their protective effects at different stages of the coping process: ability EI aids in the selection of coping strategies whereas trait EI predicts the implementation of such strategies once selected (Davis and Humphrey, 2014).

Finally, when researchers/practitioners are interested in a broader set of emotion-related and social-related dispositions and competencies we recommend a mixed measure. Mixed measures are particularly appropriate in the context of the workplace. This seems to be the case for two reasons: first, the tendency to frame EI as a set of competencies that can be trained (e.g., Goleman, 1995; Boyatzis and Goleman, 2007) is likely to equip workers with a positive growth mindset regarding their EI. Second, the emphasis on 360 degree forms of assessment in mixed measures provides individuals with information not only on their self-perceptions, but on how others perceive them which is also particularly useful in training situations.

#### Advantages and Disadvantages of Trait and Ability EI

There are numerous advantages and disadvantages of the different forms of EI that test users should factor into their decision. One disadvantage of self-report measures is that people are not always good judges of their emotion-related abilities and tendencies (Brackett et al., 2006; Sheldon et al., 2014; Boyatzis, 2018). A further disadvantage of self-report, trait based measures is their susceptibility to faking. Participants can easily come across as high in EI by answering questions in a strategic, socially desirable way. However, this is usually only an issue when test-takers believe that someone of importance (e.g., a supervisor or potential employer) will have access to their results. When it is for self-development or research, individuals are less likely to fake their answers to trait EI measures (see Tett et al., 2012). We also note that the theoretical bases of trait and mixed measures have also been questioned. Some have argued for example that self-report measures of EI measure nothing fundamentally different from the Big Five (e.g., Davies et al., 1998). We will not address this issue here as it has been extensively discussed elsewhere (e.g., Bucich and MacCann, 2019) however we emphasize that regardless of the statistical distinctiveness of self-report measures of EI, there is little question regarding their utility and predictive validity (O'Boyle et al., 2011; Miao et al., 2017).

One advantage of ability based measures is that they cannot be faked. Test-takers are told to give the answer they believe is correct, and consequently should try to obtain a score as high as possible. A further advantage is that they are often more engaging tests. Rather than simply rating agreement with statements as in trait based measures, test-takers attempt to solve emotion-related problems, solve puzzles, and rate emotions in pictures.

Overall however, there are a number of fundamental problems with ability based measures. First, many personality and intelligence theorists question the very existence of ability EI, and suggest it is nothing more than intelligence. This claim is supported by high correlations between ability EI and IQ, although some have provided evidence to the contrary (e.g., MacCann et al., 2014). Additionally, the common measures of ability EI tend to have relatively poor psychometric properties in terms of reliability and validity. Ability EI measures do not tend to strongly predict outcomes that they theoretically should predict (e.g., O'Boyle et al., 2011; Miao et al., 2017). Maul (2012) also outlines a comprehensive set of problems with the most widely used ability measure, the MSCEIT, related to consensus-based scoring, reliability, and underrepresentation of the EI construct. Also see Petrides (2011) for a comprehensive critique of ability measures.

#### General Recommendation for Non-experts Choosing Between Ability and Trait EI

While the distinction between trait, ability and mixed EI is important, we acknowledge that many readers will simply be looking for an overall measure of emotional functioning that can predict personal and professional effectiveness. Therefore, when potential users have no overt preference for trait or ability measures but need to decide, *we strongly recommend researchers/ practitioners begin with a trait-based measure of EI*. Compared to ability based measures, trait based measures tend to have very good psychometric properties, do not have questionable theoretical bases and correlate moderately and meaningfully with a broad set of outcome variables. In general, we believe that trait based measures are more appropriate for most purposes than ability based measures. That being said, several adequate measures of ability EI exist and these have been reviewed in the Literature Review section. If there is a strong preference to use ability measures of EI then several good options exist as outlined later.

### Choosing a Specific Measure of Trait EI

Based on our literature review we suggest that a very good, comprehensive measure of trait EI is the Trait Emotional Intelligence Questionnaire, or TEIQue (Petrides and Furnham, 2001). If users are not restricted by time or costs (commercial users need to pay, researchers do not) then the TEIQue is a very good option. The TEIQue is a widely used questionnaire that measures 4 factors and 15 facets of trait EI. It has been cited in more than 2,000 academic studies. It is regarded as a “trait” measure of EI because it is based entirely on self-report responses, and facet scores represent typical behavior rather than maximal performance. There is extensive evidence in support of its reliability and validity (Andrei et al., 2016). The four factors of the TEIQue map on to the broad EI facets present in multiple measures of EI as follows: emotionality = perceiving emotions, self-control = regulating emotions in self, sociability = regulating emotions in others, well-being = strategically utilizing emotions.

One disadvantage of the TEIQue however is that it is not freely available for commercial use. The website states that commercial or quasi-commercial use without permission is prohibited. The test can nevertheless be commercially used for a relatively small fee. The relevant webpage can be found here (http://psychometriclab.com/). A second disadvantage is that the test can be fairly easily faked due to its use of a self-report response scale. However, this is generally only an issue when individuals have a reason for faking (e.g., their score will be seen by someone else and might impact their prospects of being selected for a job) (see Tett et al., 2012). Consequently, we do not recommend the TEIQue to be used for personnel selection, but it is relevant for other professional purposes such as in EI training and executive coaching.

There are very few free measures of trait EI that have been adequately investigated. One exception is the widely used, freely available measure termed the Self-Report Emotional Intelligence Test (SREIT, Schutte et al., 1998). The SREIT has been cited more than 3,000 times. The full paper which includes all test items can be accessed here (https://www.researchgate.net/publication/247166550_Development_and_Validation_of_a_Measure_of_Emotional_Intelligence). Although it was designed to measure overall EI, subsequent research indicates that it performs better as a multidimensional scale measuring 4 distinct factors including: optimism/mood regulation, appraisal of emotions, social skills and utilization of emotions. These four scales again map closely to the broad facets present in many EI instruments as follows: optimism/mood regulation = regulating emotions in self, appraisal of emotions = perceiving emotions in self, social skills = regulating emotions in others, and utilization of emotions = strategically utilizing emotions. Please note that although one study has comprehensively critiqued the SREIT (Petrides and Furnham, 2000), it actually works well as a multidimensional measure. This was acknowledged by the authors of the critique and has been subsequently confirmed (e.g., by O'Connor and Athota, 2013).

### Long vs. Short Measures of Trait EI

The TEIQue is available in long form (153 items, 15 facets, 4 factors) and short form (30 items, 4 factors/subscales). A complete description of all factors and facets can be found here (http://www.psychometriclab.com/adminsdata/files/TEIQue%20interpretations.pdf). We recommend using the short form when users are interested in measuring only the 4 broad EI factors measured by this questionnaire (self-control, well-being, sociability, emotionality). Additionally, there is much more research on the short form of the questionnaire (e.g., Cooper and Petrides, 2010) (see Table 5), and the scoring instructions for the short form are freely available for researchers. If the short form is used, it is recommended that all factors/subscales are utilized because they predict outcomes in different ways (e.g., O'Connor and Brown, 2016). The SREIT is available only as a short, 33 item measure. All subscales are regarded as equally important and should be included if possible. Again it is noted that this test is freely available and the article publishing the items specifically states “Note: the authors permit free use of the scale for research and clinical purposes.”

When users require a comprehensive measure of trait EI, the long form of the TEIQue is also a good option (see Table 5). Although not as widely researched as the short version, the long version nevertheless has strong empirical support for reliability and validity. The long form is likely to be particularly useful for coaching and training purposes, because the use of 15 narrow facets allows for more focused training and intervention than measures with fewer broad facets/factors.

### Choosing Between Measures of Ability EI

The most researched and supported measure of ability EI is the Mayer, Salovey, Caruso Emotional Intelligence Test (MSCEIT) (see Tables 2, 3). It has been cited in more than 1,500 academic studies. It uses a 4 branch approach to ability EI and measures ability dimensions of perceiving emotions, facilitating thought, understanding emotions and managing emotions. These scales broadly map onto the broad constructs present in many measures of EI as follows: facilitating thought = strategically utilizing emotions, perceiving emotions = perceiving emotions in self and others, understanding emotions = understanding emotions, and managing emotions = regulating emotions in self and others. However, this is a highly commercialized test and relatively expensive to use. The test is also relatively long (141 items) and time consuming to complete (30–45 min).

A second, potentially more practical option includes two related tests of ability EI designed by MacCann and Roberts (2008) (see Tables 2, 7). These tests are called the Situational Test of Emotion Management (STEM) and the Situational Test of Emotional Understanding (the STEU). These tests are becoming increasingly used in academic articles; the original paper has now been cited more than 250 times. The two aspects of ability EI measured in these tests map neatly onto two of the broad EI constructs present in multiple measures of EI. Specifically, the STEM can be regarded as a measure of emotional regulation in oneself and the STEU can be regarded as a measure of emotional understanding. As indicated in Table 7, there is strong psychometric support for these tests (although the alpha for STEU is sometimes borderline/low). A further advantage of STEU is that it contains several items regarding workplace behavior, making it highly applicable for use in professional contexts.

If researchers/practitioners decide to use the STEM and STEU, additional measures might be required to measure the remaining broad EI constructs present in other tests. Although these measures could all come from relevant scales of tests reviewed in this article (see Table 1), there is a further option. Users should consider the Diagnostic Analysis of Non-verbal Accuracy scale (DANVA) which is a widely used, validated measure of perceiving emotion in others (see Nowicki and Duke, 1994 for an introduction to the DANVA). Alternatively, for those open to using a combination of ability and trait measures, users might wish to use Schutte et al.'s (1998) SREIT to assess remaining facets of EI (see Table 4). This is because it is free and captures aspects of EI not measured by STEM/STEU. These include appraisal of emotions (for perceiving emotions) and utilization of emotions (for strategically utilizing emotions), respectively.

**Table 1:** Summary of recommended emotional intelligence assessment measures for each broad EI construct.

**Broad EI construct**	**Best overall**	**Best free**
Perceiving emotions in self and others	Self-Perception (EQ-i)	Appraisal of emotions (SREIT)
Regulating emotions in self	Self-control (TEIQue-SF)	Optimism/mood regulation (SREIT)
Regulating emotions in others	Sociability (TEIQue-SF)	Social skills (SREIT)
Strategically utilizing emotions	Relationship management (ESCI) or emotionality (TEIQue-SF)	Utilization of emotions (SREIT)

Therefore, if there is a strong preference to utilize ability based measures, the STEM, STEU, and DANVA represent some very good options worth considering. The advantage of using these over the MSCEIT is the lower cost of these measures and the reduced test time. Although the STEM, STEU, and DANVA do not seem to be freely available for commercial use, they are nevertheless appropriate for commercial use and likely to be cheaper than alternative options at this point in time.

### Deciding Between Using a Single Measure or Multiple Measures

When seeking to measure EI, researchers/practitioners could choose to use (1) a single EI tool that measures overall EI along with common EI facets (i.e., perceiving emotions in self and others, regulating emotions in self and others and strategically utilizing emotions) or (2) some combination of existing scales from EI tool/s to cumulatively measure the four constructs.

The first option represents the most pragmatic and generally optimal solution because all information about the relevant facets and related measures would usually be located in a single document (e.g., test manual, journal article) or website. Additionally, if a paid test is used it would only require a single payment to a single author/institution. Furthermore, single EI tools are generally based on theoretical models of EI that have implications for training and development. For example EI facets in Goleman's (1995) model (as measured using the ESCI, Boyatzis and Goleman, 2007) are regarded as characteristics that can be trained. Therefore, if a single EI tool is selected, the theory underlying the tool could be used to model the interventions.

However, a disadvantage of the first option is that some EI measures will not contain the specific set of EI constructs researchers/practitioners are interested in assessing. This will often be the case when practitioners are seeking a comprehensive measure of EI but prefer a freely available measure. The second option specified above would solve this problem. However, the trade-off would be increased complexity and the absence of a single underlying theory that relates to the selected measures. Tables 2–8 describe facets within each measure as well as reliability and validity evidence for each facet and can be used to assist the selection of multiple measures if users choose to do this.

**Table 2:** Summary of major emotional Intelligence assessment measures.

**Name of tool and original citation**	**Explanation of the theoretical basis**	**Test length and description**	**Example Item/s**	**Availability free vs. cost**
Mayer-Salovey-Caruso Emotional Intelligence Test (MSCEIT) Mayer et al. (2002a,b, 2003) Cited in more than 1,500 articles	In 1997 Salovey and Mayer developed a 4 branch approach to ability EI called MEIS and since then this has been developed into the MSCEIT (Mayer et al., 2002a,b) and revised with additional versions. The revised model is a process-orientated model that emphasizes stages of development in EI, potential for growth and the contributions emotions make to intellectual growth. The scale was developed based on a review of ability EI literature around focusing on individuals' processing of emotion related information. Each of the four branches is measured with two objective, ability-based tasks. There are different response formats. Some tasks such as the “picture task,” use 5-point rating scales, whereas other tasks, such as the “blends task,” use a multiple-choice response. For all questions however, answers can be considered correct or incorrect in a similar way to IQ tests. The facets can be defined as follows: Perceiving Emotion represents the ability to correctly identify how oneself and others are feeling. Facilitating Thought represents the ability to create emotions that impact thought processes. Understanding Emotion represents the ability to understand the causes of emotions. Managing Emotion represents the ability to create effective strategies that utilize emotions for a specific purpose.	Consists of 8 MSCEIT tasks which are made up of a number of individual items. 141 questions in total. 4 constructs including: Perceiving Emotions; Facilitating Thought; Understanding Emotions; Managing Emotions.	In the faces task (four item parcels; 5 responses each), participants view a series of faces and for each, respond on a five-point scale, indicating the degree to which a specific emotion is present in the face.	Cost Website https://www.mhs.com/
Self-report Emotional Intelligence Test (SREIT) Schutte et al. (1998) Cited in more than 3,000 articles	Schutte et al. (1998) developed a self-report EI questionnaire based on Salovey and Mayer's (1990) model. A factor analysis was conducted on 62 items using data from 346 participants from which a 33-item scale was created. The measure showed good internal consistency (Cronbach's alpha of 0.90) and test-retest reliability (*r* = 0.78). The scale was also tested against theoretically related constructs including alexithymia, non-verbal communication of affect, optimism, pessimism, attention to feelings, clarity of feelings, mood repair, depressed mood and impulsivity and found to have construct validity. The model however has been criticized for confusing ability and trait forms of EI (however this criticism can be applied to the development of most trait based models). Participants respond to items on a 5-point Likert-type scale ranging from 1 (strongly-disagree) to 5 (strongly-agree).	Consists of 33 self-report statements. Four factors including: 1. Optimism/ mood regulation 2. Appraisal of emotions 3. Social skills 4. Utilization of emotions	An example item is “I am aware of my emotions as I experience them”.	Free
Trait Emotional Intelligence Questionnaire (TEIQue). Long Form and Short Forms. Petrides and Furnham (2001) Cited in more than 2,000 articles	**TEIQue-Long Form** The TEIQue is based on trait EI theory, which conceptualizes emotional intelligence as a personality trait. It has also been described as “emotional self-efficacy.” Unlike Schutte et al.'s (1998) measure, it did not originally aim to measure ability based EI with self-report questions. Item and facets were developed by conducting a content analysis of the EI literature and available constructs (Salovey and Mayer, 1990; Goleman, 1995; Bar-On, 1997a,b). **TEIQue-Short Form** Petrides and Furnham also created a short-form questionnaire (TEIQue-SF) which contains 30 items and the same 4 factors from the long version. Additional adaptations such as a 360 degree measure can be found on their website http://psychometriclab.com/	Consists of 153 self-report statements. Four factors and 15 facets including: 1.Well-being (Trait optimism, trait happiness and self-esteem); 2. Sociability (Emotional management (others), assertiveness and social awareness); 3. Emotionality (trait empathy, emotional perception, emotion expression and relationships); 4. Self-control (emotion regulation, impulsiveness and stress management).	Example items include: “Understanding the needs and desires of others is not a problem for me”; “I'm usually able to influence the way other people feel” and “I can handle most difficulties in my life in a cool and composed manner.”	Cost. Not freely available for commercial use. Details for obtaining permission are on website. Free for research purposes.
Bar-On Emotional Quotient Inventory (EQ-i) Bar-On (1996, 1997a,b) Cited in more than 1,000 articles	Mixed position, considers EI as a mixed construct consisting of both cognitive ability and personality aspects. The scale emphasizes how the personality traits influence a person's general well-being. Bar-On's model was based on empirical research into personal factors related to EI and particularly into emotional and social elements of behavior. The concept was theoretically developed from logically clustering variables and identifying underlying key factors claimed to determine effective and successful functioning. The EQ-i measures abilities and the potential for performance rather than performance itself; it is process-oriented, rather than outcome-oriented. Bar-On's original report of EQ-i from 1996 is in a book form. However, since the development of the original EQ-i scale, Bar-On and others have revised the scale (Bar-On et al., 2000) thus creating EQ-i 2.0. The total EQ-i can be used to create total EI scores as well as factor and facet/subscale scores. The subscales have adequate internal consistency. Bar-On went on to develop additional test versions including a youth version (EQ-i:YV) and 360 multi-rater measure (EQ-360).	Revised model consists of 125 items. Five factors, 15 facets (subscales) including: 1. Self-Perception (Self-Regard, Self-Actualization, Emotional Self Awareness) 2. Interpersonal (Interpersonal Relationships, Empathy Social Responsibility) 3. Decision Making (Problem Solving, Reality Testing, Impulse Control) 4. Self-Expression (Emotional Expression, Assertiveness Independence) 5. Stress Management (Flexibility Stress Tolerance, Optimism)	Example items include: “When I'm angry with others, I can tell them about it,” “I know how to deal with upsetting problems,” and “I like helping people.”	Cost http://www.reuvenbaron.org/wp/
The situational test of emotion management (STEM). The situational test of emotional understanding (STEU) MacCann and Roberts (2008) Cited in more than 250 articles	MacCann and Roberts (2008) developed ability-based measures of EI. MacCann and Roberts based their STEM and STEU scales on 2 of the four hierarchical ordered branches of emotion-related abilities outlined by Mayer et al. (2000): understanding and managing emotions which form the Strategic EI area (Mayer et al., 2001). **STEM** The STEM was developed to be administered in both multiple-choice and rate-the-extent formats (i.e., test takers rate the appropriateness, strength, or extent of each alternative, rather than selecting the correct alternative). Items for STEM were developed by conducting semi-structured interviews with 50 individuals who described emotional situations they experienced in the past 2 weeks (with a total of 290 situations). These items were categorized and tested. **STEU** Roseman's (2001) emotion appraisal theory was used as the basis for item construction and scoring of the STEU such that answers could be regarded as correct or incorrect. According to this model, the 17 most common emotions can be explained by a combination of seven appraisal dimensions. The STEU comprised 42 items with each item presenting emotional situations, and participants had to choose which emotion the situation will most likely elicit. Fourteen emotions were assessed in 3 separate contexts—de-contextualized, work and private life.	STEM−44 items Anger (18 items); sadness (14 items) and fear (12 items). STEU −42 items (14 context-reduced, 14 with a personal-life context, and 14 with a workplace context.	STEU—workplace example assessing relief includes: a supervisor who is unpleasant to work for leaves Alfonso's work. Alfonso is most likely to feel? (a) joy, (b) hope, (c) regret, (d) relief, (e) sadness	Can be freely obtained in Appendix 2.1 of MacCann's (2006) study. However permission is required to use the test for non-research purposes.
Emotional and Social Competence Inventory (ESCI). Boyatzis et al. (2000) Cited in more than 1,500 articles	The ESCI is based on a mixed model of EI and regards EI as consisting of both cognitive ability and personality aspects. The model focuses heavily on predicting workplace success. The ESCI utilizes 360 degree assessment that can include self-ratings, peer ratings and supervisor ratings. Boyatzis and Goleman include a set of emotional competencies within each construct of EI. Emotional competencies are not regarded as innate talents, but rather learned capabilities that must be worked on and can be developed to achieve outstanding performance. Boyatzis and Goleman argue that individuals are born with a general emotional intelligence potential that determines their potential for learning emotional competencies. Internal consistency of the scales ranges from 0.61 to 0.85 (Conte, 2005).	Consists of 110 items Assesses 12 competencies organized into four factors: 1.Self awareness 2. Social awareness 3. Self-Management 4. Relationship management	Example items includes: “I recognize my emotions and their effects on others,” and “I can keep disruptive emptions or impulses under control.”	Cost

*Note the measures reviewed above were selected based on widespread use and validation. Although other measures exist, they were not reviewed based on either less research in general or poor psychometric support. However, if none of those reviewed above are considered appropriate, three further available measures could be considered. One relatively new measure with good preliminary support is the Genos Emotional Intelligence Inventory (Palmer et al., 2009). This is a commercial, mixed measure of EI and requires payment. A further, freely available measure is Wong's Emotional Intelligence Scale (WEIS) (trait-based; see Wong et al., 2004, 2007). A third very new measure is the Geneva Emotional Competence Test (GECo) (see Schlegel and Mortillaro, 2019). It is an ability based measure designed for the workplace that looks very promising based on early work*.

**Table 3:** Review of selected studies detailing psychometric properties of the Mayer-Salovey-Caruso Emotional Intelligence Test (MSCEIT).

**Citation and country**	**Participants (*N*, age, occupation, gender ratio etc.)**	**Study design**	**Reliability and validity evidence**	**Comment**
Mayer et al. (2002a,b, 2003) Multiple countries, primarily USA sample.	*Non clinical* *N* = 2,112 *Sample:* individuals from academic settings across multiple countries. *Age:* mean age was 26.25 years. Gender: 58.6% female.	The design was cross sectional: participants completed the MSCEIT. The test was administered via booklet or online. Scoring was based on how well-respondents' answers aligned with an expert sample (volunteer members of the International Society for Research on Emotions).	**Internal consistency:** Mayer et al. (2002b) reported reliabilities of 0.91 for the full scale, 0.81 for emotional management, 0.77 for emotional understanding, 0.76 for emotional facilitation, and 0.90 for emotional perception. **Test-retest reliability:** Mayer et al. (2003) reported a test-retest reliability of 86 and a full-test split-half reliability of 0.93.	
Brackett and Mayer (2003) USA	*Non clinical* *N* = 207 *Sample:* University students. *Gender:* 130 female, 77 male. *Age:* Mean age was 18.93 for females and 19.51 for males.	The study aimed to investigate the convergent, discriminant and incremental validity of the MSCEIT as well as two other EI measures (EQ-i and SREIT). Participants completed a self-assessed questionnaire and were assessed on their psychological well-being, personality, subjective well-being and academic ability.	**Internal consistency:** The authors did not report values for internal consistent from this study but rather cited the values from Mayer et al. (2002). **Validity:** The MSCEIT had discriminant validity against well-studied personality and well-being measures. Additionally, after personality and verbal intelligence were held constant, the MSCEIT was predictive of social deviance. MSCEIT scores were also found to relate positively and significantly to psychological well-being (*r* = 0.28, *p* < 0.001), agreeableness (*r* = 0.28, *p* < 0.001) and openness (*r* = 0.25, *p* < 0.001).	The generalizability of the findings to the general working population may be limited due to the student sample.
Rosete and Ciarrochi (2005) Australia	*Non clinical* *N* = 41 *Sample:* Executives from a large Australian Public Service organization *Gender*: 24 male and 18 female. *Age*: Age ranged from 27 to 57 with an average age of 42 years. *Tenure*: 75% of participants had been in the organization for 10 years or more.	Participants were sought from the organization to participate in a career development exercise. Questionnaires were completed via pen or paper or online. Participants completed the MSCEIT (V2.0), along with a measure of personality traits, and a measure of cognitive ability. Leadership effectiveness was assessed using an objective measure of performance and a 360 degree assessment involving each leader's subordinates and direct manager (*n* = 149).	**Internal consistency:** The authors did not report values for internal consistency from this study but rather cited the values from Mayer et al. (2002). **Construct validity:** The study found that scores from the MSCEIT were correlated with cognitive intelligence, specifically verbal IQ (*r* = 0.336, *p* < 0.05); performance IQ (*r* = 0.402, *p* < 0.05), and full scale IQ (*r* = 0.430, *p* < 0.01).	The findings should be generalized with caution due to the small sample size and one industry sampled. Similarly, the executives in this study had significantly higher IQs than the average population which could also limit the generalizability of results.
Ruiz-Aranda et al. (2014) Spain	*Non clinical* *N* = 264 *Sample:* University students from the School of Health and Science—Specifically, students studying nursing, physiotherapy, occupational therapy and chiropody. *Gender:* All female. *Age:* Ages ranged from 18 to 50 with a mean age of 21 years.	Participants completed a Spanish version of the MSCEIT along with measures of well-being (life-satisfaction and happiness) and perceived stress.	**Internal consistency:** Total score Cronbach's alpha was 0.76. **Construct validity:** Higher EI scores were found to be related to lower levels of perceived stress and higher levels of life satisfaction and happiness.	The sample was made up of exclusively females, which means that the results obtained my not be generalizable to the male population. The authors also suggest the study did not control for personality which may have an impact on the results.

*Note two of the studies reviewed in this table utilize student samples. As specified in the inclusion criteria section we targeted non-student samples and only utilized student samples where others were not available or not appropriate*.

**Table 4:** Review of selected studies detailing psychometric properties of the Self-report Emotional Intelligence Test (SREIT).

**Citation and country**	**Participants (*N*, age, occupation, gender ratio etc.)**	**Study design**	**Reliability and validity evidence**	**Comment**
Schutte et al. (1998) USA	*Non clinical* *N* = 346 Sample: University students and individuals from diverse community settings. Gender: 218 were women and 111 were men. Age: average age was 29.27 years.	Self-assessment questionnaire. All 346 participants rated themselves on the 62 EI items, with a number of participants also filling out one of several established scales to measure constructs theoretically related to EI. Additional scales included; an alexithymia scale which assessed difficulties in identifying and describing feelings; a communication test which assessed non-verbal expressiveness; a life orientation test which assessed optimism and pessimism; a mood scale including assessing attention to feelings and mood repair; a scale to measure depressed mood; and a measure of impulsiveness.	**Internal consistency:** Cronbach's alpha of 0.90 was obtained. **Test retest reliability:** 28 students repeated the test 2 weeks later, with a test-retest reliability of 0.78. **Construct validity:** validation studies showed that scores on the 33-item measure correlated with eight of nine theoretically related constructs, including, alexithymia (*r* = −0.65, *p* < 0.0001), attention to feelings (*r* = 0.63, *p* < 0.0001), clarity of feelings (*r* = 0.52, *p* < 0.0001), mood repair (*r* = 0.68, *p* < 0.0001), optimism (*r* = 0.52, *p* < 0.006), pessimism (*r* = −0.42, *p* < 0.025), depression (*r* = −0.37, *p* < 0.021) and impulse control (*r* = −0.39, *p* < 0.003). **Predictive validity:** a test was conducted on college students to assess the predictive validity. Results revealed that the EI measure completed at the start of the academic year, significantly predicted first year college grades at the end of the year (*r* = 0.32, *p* < 0.01). **Discriminant validity:** Scores on the measure were tested against the big five personality dimensions to assess discriminant validity and were only associated with the openness to experience trait.	
Kinman and Grant (2011) UK	*Non clinical* *N* = 240 Sample: Trainee social work students (69% of the sample were first-year students and 31% second-year students). Gender: 82% female Age: Mean age of 33.7 years.	Self-report questionnaire. Participants were invited to participate via email and competed the questionnaire online. The aim of the study was to explore the role of emotional and social competencies on resilience. The study also assessed measurements of reflective ability, empathy, social competence, resilience and psychological distress.	**Internal consistency:** Cronbach's alpha was 0.88. **Construct validity:** Emotional Intelligence was correlated to additional measures as expected. For example EI was positively correlated with resilience (*r* = 0.61, *p* < 0.001), reflective ability (*r* = 0.59, *p* < 0.001), and empathy (*r* = 0.45, *p* < 0.001),	The study is based on a cross-sectional and correlational data. Although some of the relationships found between emotional and social competencies and resilience and well-being were strong, cause and effect cannot be established using such methodology.
Por et al. (2011) UK	*Non clinical* *N* = 130 Sample: Student nurses. Gender: 117 female, 13 male. Age: Mean age of 28 years.	Data was collected through self-report questionnaire, an audit of students' academic performance and mapping of EI teaching material. The study aimed to explore the emotional intelligence of nursing students and its relationship to perceived stress, coping strategies, subjective well-being, perceived nursing competency and academic performance.	**Internal consistency:** Cronbach's alpha was 0.82. **Construct validity:** There was a strong negative correlation between EI and perceived stress (*r* = −0.40, *p* < 0.01). EI was positively related to perceived nursing competency (*r* = 0.32, *p* < 0.01) and subjective well-being (*r* = 0.27, *p* < 0.01).	There are some limitations to the study such as the small sample size and the fact that the study only involved students that may limit the generalizability to other occupations. Data being collected at a single point in time means that potential changes in participants over time were not captured.

**Table 5:** Review of selected studies on psychometric properties of the Trait Emotional Intelligence Questionnaire (TEIQue).

**Citation and Country**	**Participants (*N*, age, occupation, gender ratio etc.)**	**Study design**	**Reliability and validity evidence**	**Comment**
Petrides (2009) Primarily UK participants	*Non clinical* *N* = 1724 Sample: mixed normative sample Gender: 912 females, 764 females, 61 unreported. Mean age: 29.65	The statistics provided in this paper were based on the full norm sample of the TEIQue at the time of publication. The study design is best regarded as cross sectional, with all participants having completed the TEIQue. Data from 58 students was presented for test-retest reliability.	**Internal consistency:** Cronbach's alpha for the global trait EI score was 0.89. Alpha for the 15 facets and 4 factors ranged from 0.67 to 0.92. **Test-retest reliability:** This was provided for the four factors (Emotionality, Self-control, Sociability, Well-being) and ranged from 0.59 to 0.86. **Construct validity:** Some evidence for construct validity was provided based on exploratory factor analysis. **Self-other ratings:** Self-other ratings were significant for global EI (*r* = 0.48) and ranged from 0.29 to 0.52 across the 15 facets and 4 factors.	This study was published as book chapter and is freely available to access online^1^.
Mikolajczak et al. (2007) Short form Belgium	*Non clinical* N = 124 Sample: Nurses Gender: 85% female, 15% male Mean age: 39.4 years	This study used the TEIQue Short form survey to understand the relationship between trait emotional intelligence and occupational stress. Participants completed two separate surveys 3 months apart. Trait EI, the Big Five personality traits and emotional labor style were assessed at Time 1, whereas burnout and somatic complaints were measured at both T1 and T2.	**Internal consistency:** Cronbach's alpha for the TEIQue-SF scale was recorded as 0.87. **Construct validity:** Trait EI was correlated with a number of other constructs such as global burnout (*r* = −0.58, *p* < 0.001), diminished accomplishment (*r* = −0.64, *p* < 0.001), and emotional exhaustion (*r* = −0.49, *p* < 0.001). **Incremental validity:** Incremental validity was tested using hierarchical regression controlling for the Big Five personality traits. Trait EI demonstrated incremental validity over and above the Big Five for a number of outcomes.	Only self-report measures used.
Cooper and Petrides (2010) Short form UK	*Non clinical* Study 1: *N* = 1,119 Sample: University students and general community. Gender: 455 males and 653 females. Age: Age ranged from 15 to 89 years with an average age of 32 years. Education: high school diplomas (21%), undergraduate diplomas (41%), postgraduate diplomas (33%) and PhD (2%). Study 2: *N* = 866 Sample: University students and general community. Gender: 432 males and 416 females. Age: Age ranged from 17 to 80, with an average age of 27 years. Education: high school diplomas (20%), undergraduate diplomas (41%), postgraduate diplomas (26%) and PhD (3%).	The aim of the research was to conduct psychometric analysis on the TEIQue-SF and create a revised model. Study 1: Individuals were recruited via word of mouth, advertisement through social media, and course data collection. The 30-item TEIQue version 1 was administered with a pen and paper questionnaire. Confirmatory factor analysis was conducted and four of the items were re-written. Study 2: The students completed version 1.5 of the TEIQue developed in study 1. The same procedure was carried out in study 2.	**Internal consistency:** In study 1 (TEIQue –SF), Cronbach's alpha for men was 0.89 and 0.88 for women. In study 2 (TEIQue-SF 1.50), Cronbach's alpha for men was 0.88 and 0.87 for women. **Construct validity:** Each measure was tested using item response theory (IRT) which provides information about measurement precision for each item. Taken together, the results of the IRT analysis suggest TEIQue-SF has good psychometric properties	
Heffernan et al. (2010) Short form USA	*Non clinical* *N* = 135 Sample: RN nurses (34% medical unit, 12% surgery and 12% critical care). Gender: 95% of participants were female. Age: 34% of nurses were aged 41–50 and 31% were aged 52–60. Education: 42% bachelor level and 28% masters level.	The study assessed self-compassion and emotional intelligence using the TEIQue -SF in nurses. Nurses completed the self-report assessment online.	**Internal consistency:** Cronbach's alpha of 0.88 was reported for the study. **Construct validity:** The study found EI was significantly related to self-compassion (*r* = 0.55, *p* < 0.0001).	

Note some of the studies reviewed in this table utilize student samples. As specified in the inclusion criteria section we targeted non-student samples and only utilized student samples where others were not available or not appropriate.

1 http://www.psychometriclab.com/adminsdata/files/TEIQue%20psychometric%20properties%20chapter.PDF

**Table 6:** Review of selected studies on psychometric properties of the Emotional Quotient Inventory (EQ-i) (Bar-On, 1997a,b).

**Citation and Country**	**Participants (*N*, age, occupation, gender ratio etc.)**	**Study Design**	**Reliability and Validity Evidence**	**Comment**
Bar-On et al. (2000); Dulewicz et al. (2003); and Bar-On (2006) USA and Canada	*Non clinical* *N* = 3,831 Gender: 49% male, 51% female. Sample: varied occupations. Age: Age ranged from 16 to 100, with an average age of 34.3 years. Ethnicity: The sample was 79% White, 8% Asian American, 7% African American, 3% Hispanic, and 1% Native American.	The EQ-I has been developed over 17 years by Bar-On. Numerous studies have been conducted by Bar-On testing the self-report measure to establish a valid and reliable tool. Many of his earlier works were not able to be located however information was drawn from a number or sources listed to the left.	**Internal consistency:** The overall internal consistency was reported at 0.97. **Test-retest reliability:** the average stability coefficient is 0.85 after 1 month and 0.75 after 4 months. **Predictive validity:** Bar-On (2006) noted 20 predictive validity studies that have been conducted on a total of 22,971 individuals across 7 counties. The EQ-i measure was found to predict performance in social interactions, at school and work as well as impacts on physical health, psychological health, self-actualization and subjective well-being. The average predictive validity coefficient is 0.59.	
Bar-On et al. (2000) Germany	*Non clinical* *N* = 167 Sample: Helping professionals including police officers (*n* = 85) and child care and mental health care workers (*n* = 81). Gender: 72% male and 28% female. Age: mean age was 33.2 years. Education: the average duration of education was 11.9 years.	Self-assessment questionnaire. Used the earlier version of Bar-On's EQ-i comprising of 133 items translated to German. The study assessed occupational stress and emotional expression within different high stress helping professions, namely the police force and child care and mental health care professions. The authors examined gender, age and occupational differences.	**Internal consistency:** Alpha coefficients ranged from 0.66 to 0.87 for the scales.	The authors noted that there may be social desirability bias present. Specific organizational stressors were not assessed in the study therefore there organizational or occupational differences may be present. Results may not be generalizable to the wider population due to the limited sample size. Cross-sectional study – a longitudinal study is required to assess causality. Self-report measure—this study relies on subjective self-report data.
Dawda and Hart (2000) Canada	*Non clinical* *N* = 243 Sample: University students Gender: 118 men and 125 women Age: Age ranged from 17 to 47 with a mean age of 21.27 years.	Students were recruited via posters advertising an “emotions study.” The aim of the research was to assess the validity and reliability of the EQ-i measure, and was undertaken as part of a larger study examining the association between psychopathy and alexithymia. Participants completed the EQ-i measure, as well as two interview-based rating scales for alexithymia, and a range of self-report measures including alexithymia, personality, affect intensity, depression and psychosomatic complaints.	**Internal consistency:** Cronbach's alpha for the full scale was 0.96 with coefficients ranging from 0.81 to 0.94 for the factors. **Construct validity:** The correlations between EI and the additional scales generally were moderate, ranging from 0.32 to 0.83. In general, people with high EQ Total scores had low levels of negative affectivity and high levels of positive affectivity; were conscientious and agreeable; had fewer difficulties identifying and describing feelings; and were not prone to somatic symptomatology or increased somatic symptoms under stress.	One concern was that the Interpersonal scale had relatively small correlations with the other EQ composite scales, as well as a different pattern of convergent and discriminant validities. The authors were unable to explain below-normal EQ-i scores in the study however the low scores should not have much impact on the observed convergent/discriminant validity. For specific aspects of EI, the authors suggest to use the 15 subscale scores instead of the 5 factors, which are generally more internally consistent.

*Note some of the studies reviewed in this table utilize student samples. As specified in the inclusion criteria section we targeted non-student samples and only utilized student samples where others were not available or not appropriate*.

**Table 7:** Review of selected studies on psychometric properties of the STEU and STEM.

**Citation and country**	**Participants (*N*, age, occupation, gender ratio etc.)**	**Study design**	**Reliability and validity evidence**	**Critique/limitations**
STEU and STEM MacCann and Roberts (2008) Australia	*Non clinical* Study 1: *N* = 207 Sample: Psychology undergraduate students. Participants were drawn from both a rural campus and urban campus of Sydney University. Gender: 140 female. Age: Average age was 21.1 years. Study 2: *N* = 149 Sample: Volunteers recruited from the Sydney area via advertising. Gender: 107 females. Age: Aged 18–59 with an average age of 35 years. Education: 68% of the sample had postsecondary school qualifications.	Study 1—Quasi-experimental design using self-rated scenario questionnaires in which 2 groups of participants completed two different tests. Three emotion-related criteria were also used in the study including alexithymia, life satisfaction and academic achievement. Additional measures were used to test the validity and reliability of the scale including personality and depression and anxiety. Items for the STEU were developed using Roseman's (2001) emotional appraisal theory. Items for STEM were developed through semi-structured interviews assessing emotional situations individuals had recently experienced. The items were then tested on 2 groups: undergraduate students and a community sample.	**Internal consistency:** Study 1—Cronbach's alpha for the STEU was 0.71, STEM (multiple choice) was 0.68 and STEM (rate the extent) was 0.92. Study 2—Cronbach's alpha for the STEU was 0.42 and STEM (30 item) was 0.61. **Construct validity:** Relationships were established between STEU/STEM and vocabulary and university grades. Study 2—The STEU correlated with Anxiety (*r* = −0.25, *p* < 0.01) and Stress (*r* = −0.17, *p* < 0.05), but not with Depression (*r* = −0.15, ns). The STEM correlated with Anxiety (*r* = −0.27, *p* < 0.01), Stress (*r* = −0.26, *p* < 0.01), and Depression (*r* = −0.17, *p* < 0.05). **Predictive validity:** Both the STEU and STEM incrementally predicted students' psychology grades, and the STEU also incrementally predicted students' overall grades.	The validation had some issues. Further validation of the measures is need such as against the full MSCEIT scale. The author suggests that a video or audio based version (rather than text) would also be useful to determine whether relationships of EI to intelligence are due to cognitive processing of emotional information rather than to the verbal ability required to comprehend the text-based items.
Austin (2010) UK	*Non clinical* *N* = 339 Sample: Undergraduate students Gender: 238 females, 101 males. Age: Average age was 21.96 years.	The aim of the research was to assess the STEM and STEU measures against other ability measures such as MSCEIT. Participants were recruited via a website advertising research participation. Participant were divided into 2 groups (G1 = 104; G2 = 135) and completed a number of different EI ability measures with group 1 also being assessed on the TEIQue EI trait measure.	**Internal consistency:** Cronbach's alpha for STEM was 0.67 and 0.48 for STEU.	The study used an undergraduate student sample therefore generalizability to the working population may be limited. The reliability for the STEM was considered adequate however the reliability for the STEU was quite low, especially when compared to the MSCEIT Cronbach's alpha of 0.90 shown in the study.
Grant (2013) USA	*Non clinical* *N* = 100 Sample: employees at an optometry company headquarters. Positions: managers (25%), patient services representatives (19%), optical consultants and sales representatives (18%), technicians (17%), doctors (14%) and administration staff (7%). Gender: 77% were female. Age: average age was 33 years. Tenure: average tenure in the organization was 4.21 years and in their current position of 3.95 years. Education: The majority had attended college (71%) and the remaining employees had attended high school (14%) or graduate school (15%).	Self-report questionnaire design. Emails were sent to all 209 full-time employees which provided a link to an initial survey containing self-report measures of emotional labor strategies and personality traits. Once completed a second survey was sent assessing emotion regulation (EI) knowledge (on average completed 3 weeks later). Employees were assessed on their emotional regulation knowledge (measured by STEM), as well as measures such as emotional labor strategies, voice and performance evaluation, helping and extraversion.	**Internal consistency:** Cronbach's alpha for the STEM was reported at 0.73.	Due to the correlational nature of the study, it makes it difficult to rule out alternative explanations for the relationships or to predict causality. Additionally, because the employees were tested for their emotional regulation knowledge (STEM) after the other constructs, this may influence the causality direction or relationship. Contextual factors were also not measured in the study that may impact the emotional regulation knowledge and strategies. Self-report measure.

*Note some of the studies reviewed in this table utilize student samples. As specified in the inclusion criteria section we targeted non-student samples and only utilized student samples where others were not available or not appropriate*.

**Table 8:** Review of selected studies on psychometric properties of the Emotional and Social competence Inventory (ESCI).

**Citation**	**Participants (*N*, age, occupation, gender ratio etc.)**	**Study design**	**Reliability and validity evidence**	**Comment**
Technical Manual for Emotional and Social Competence Inventory (ESCI) (Boyatzis and Goleman, 2007—manual updated 2011) US and UK	*Non clinical* The manual reported the development of the ECI and ESCI utilizing 3 primary samples: *N* > 4,000 self-assessments from managers and professionals, and more than 10,000 other assessments (1998). *N* = 116 self-assessments, 1,022 other assessments (2007). *N* = 5,700 self-assessments and 62,000 other assessments (2,010).	The studies reported in the technical manual relate primarily to the factor structure, reliability and validity of the ESCI. The factor structure and reliability studies utilize a cross sectional design. Validity studies comprised a combination of self-report and other-reports, including other reports of performance (e.g., supervisor ratings).	**Internal consistency:** The internal consistency of the 12 scales of the ESCI range from 0.79 to 0.91 (*n* = 52,363; published in technical manual as well as Boyatzis and Gaskin, 2010). This is for the “other” assessments. **Test-retest reliability** data was only given from the ECI, this ranged from 0.41 to 0.92. **Construct validity:** This was assessed by examining correlations with similar constructs from the MBT (*N* = 18 paramedics). MBTI intuiting types scored highly on several EI competencies including emotional self-awareness and self-control. MBTI feeling types scored highly on self-awareness, empathy and others. Evidence for **discriminant validity** was based on low correlations with subtests of the Watson-Glaser Critical Thinking Appraisal (*N* = 90 executives). Evidence for **predictive validity** was found for self-reports of job success, life success and salary across a range of sectors *N* < 300.	The technical manual is available online^1^. It reports a combination of data from published industry reports as well as published peer-reviewed academic articles.
Morrison (2008) USA	*Non clinical* *N* = 92 Sample: registered nurses from 3 healthcare facilities in South Mississippi. Gender: of the 92 participants, 71 were female and 21 were male. Age: age ranged from 20 to over 60 years, with 47.8% of participants between 20 and 30 years of age. Ethnicity: the majority of the nurses were Caucasian (85.9%). Education: 72.8% had a Bachelor degree in nursing. Experience: over half of the nurses had four or less years of work experience.	Cross-sectional correlational design completed by both the participant and peer reviewer. The purpose of this study was to determine if a relationship exists between emotional intelligence (EI) and preferred conflict-handling styles of registered nurses. Each participant completed the ECI 2.0 (later renamed to ESCI) as well as an instrument to measure conflict handling. The participants handed a second ECI 2.0 instrument to a known manager, peer or subordinate they had worked with in the past year. The peer, subordinate or supervisor was asked to evaluate the participant using the ECI 2.0 instrument.	**Internal Consistency:** The authors reported existing Cronbach's alpha scores. Cronbach's alpha for others-rating ranged from 0.73 (Trustworthiness) to 0.92 (Empathy), with an overall (other-rating) average internal consistency coefficient of 0.85. The internal consistency coefficients for self-rating ranged from 0.61 (accurate self-assessment) to 0.85 (service orientation), with an overall average internal consistency coefficient of 0.75. **Construct Validity:** The study indicated a positive and statistically significant relationship between collaboration and all four of the EI clusters: self-awareness (*r* = 0.25), self-management (*r* = 0.32), social awareness (*r* = 0.31), and relationship management (*r* = 0.37).	Small sample size which could limit the generalizability. The authors also noted that an organizational climate survey could be administered to assess whether the organizational climate affects how a registered nurse responds when faced with conflict.
Reed et al. (2015) USA	*Non Clinical* *N* = 40 Sample: First year pediatric and medicine/pediatrics interns from a Children's Hospital. Gender: 32 females and 8 males.	Cross-sectional, self-report and peer-assessment design. The aim of the study was to determine: (1) performance of first-year pediatric residents in the delivery of bad news in a standardized patient (SP) setting; and (2) the role of EI in these assessments. Skill in bad news delivery was assessed via SP encounters using a previously published assessment tool and being exposure to a scenario. Residents completed the ESCI as a measure of EI. The ESCI was administered via a self-assessment to residents online, with eight relevant peers/supervisors invited to complete a confidential assessment of the resident. For each resident, a minimum of five other assessments were obtained and averaged to create the *Other* assessment. The *Other* assessment score was used for analysis.	**Internal Consistency**: The authors noted that the internal consistency of each ESCI subscale for *Other* assessments (i.e., peer ratings) was consistently high (Cronbach's alpha was not reported). **Construct Validity:** No ESCI subscales were significantly associated with residents' death notification skills, demonstrating no construct validity.	Limitations of this study include a small sample size drawn from a single institution, the use of a single SP encounter, and a reliance on only one type of bad news scenario (i.e., death notification) which is arguably among the most difficult. The study did not account for differences in trainees' previous experiences and/or training in breaking bad news and death notification. Additionally, limited reliability and validity data were obtained.
Boyatzis et al. (2017) USA and Northern Europe	*Non clinical* *N* = 40 Sample: Engineers in a multi-national manufacturing company. Gender: 37 males, 3 females. Age: Age ranged from 25 to 64 years with the modal age range of 35 to 44. Employment: The average tenure in the organization was 13 years. Country: 33 were from USA and 7 from Europe.	Cross-sectional, self-report and peer-assessment design. The survey was administered online. The total number of peers completing the surveys for the 40 engineers was 168 (average of 4.2 per person). Peers reported on the perceived emotional intelligence of the participant using the ESCI as well as perceived effectiveness measured with the Reputational Effectiveness Scale (RES). Self-report measures included job engagement, cognitive intelligence, personality and quality of relationships.	**Internal Consistency**: Coefficient of 0.95. This was based on the overall ESCI score since subscale scores were not used in this study. **Construct Validity:** ECI correlated with engineer reputational effectiveness (*r* = 0.70, *p* < 0.01) but not with general mental ability or personality.	This study presents a number of limitations. There was a small sample size which may limit the generalizability of the findings. The low response rate (5% valid responses) may have resulted in more of a volunteer bias than is often encountered in survey research in organizations. Further, due to the limited sample, it may be possible that the findings may be a function of organizational culture. Statistically speaking, the ESCI was completed by subordinates, so there could be an inflated effect due to common source.

1 http://www.eiconsortium.org/pdf/ESCI_user_guide.pdf

### The Best Measure of Each Broad EI Construct (Evaluated Across all Reviewed Tests)

In some cases, researchers/practitioners will not need to measure overall EI, but instead seek to measure a single dimension of EI (e.g., emotion perception, emotion management etc.). In general, we caution the selective use of individual EI scales and recommend that users habitually measure and control for EI facets they are not directly interested in. Nevertheless, we acknowledge that in some cases users will have to select a single measure and consequently, this section specifies a selection of what we consider the “best” measures for each construct. We do this for both free measures and those requiring payment. In order to determine which measure constitutes the “best” measure for each construct, the following criteria were applied:

1. The measure should have been used in multiple research studies published in high quality journals.
2. There should be good evidence for the reliability of the measure in multiple academic studies incorporating the measure.
3. The measure should have obtained adequate validity evidence in multiple academic studies. Most importantly, evidence of construct validity should have been established, including findings demonstrating that the measure correlates meaningfully with measures of related constructs.
4. The measure should be based on a strong and well-supported theory of EI.
5. The measure should be practical (i.e., easy to administer, quickly completed and scored).

Where multiple measures met the above criteria, they were compared on their performance on each criterion (i.e., a measure with a lot of research scored higher on the first criteria than a measure with a medium level of research). Table 1 summarizes these results.

Please note that the Emotional and Social Intelligence Inventory (ESCI) by Boyatzis and Goleman (2007) has subscales that are also closely related to the ones listed in Table 1 (see full technical manual here (http://www.eiconsortium.org/pdf/ESCI_user_guide.pdf). The measure was developed primarily to predict and enhance performance at work and items are generally written to reflect workplace scenarios. Subscales from this test were not consistently chosen as the “best” measures because it has not had as extensive published research as the other tests. Most research using this measure has also used peer-ratings rather than self-ratings which makes it difficult to compare with the majority of measures (this is not a weakness though). Nevertheless, it should be considered if cost is not an issue and there is a strong desire to utilize a test specifically developed for the workplace.

### Qualifications and Training

Although our purpose in this paper is not to outline the necessary training or qualifications required to administer the set of tests/questionnaires reviewed, we feel it is important to make some comments on this. First, we recommend that all researchers and practitioners considering using one more of these tests have a good understanding of the principles of psychological assessment. Users should understand the concepts of reliability, validity and the role of norms in psychological testing. There are many good introductory texts in this area (e.g., Kaplan and Saccuzzo, 2017). Furthermore, we recommend users have a good understanding of the limitations of psychological testing and assessment. When using EI measures to evaluate suitability of job applicants, these measures should form only part of the assessment process and should not be regarded as comprehensive information about applicants. Finally, some of the tests outlined in this review require specific certification and/or qualifications. Certification and/or qualification is required for administrators of the ESCI, MSCEIT, and EQi 2.0).

## Literature Review

The final section of this article is a literature review of the 6 popular measures we have covered. We have included our review at the end of this article because we regard it as optional reading. We suggest that this section will be useful primarily for those seeking a more in depth understanding of the key studies underlying the various measures we have presented in earlier sections.

This literature review had two related aims; first to identify prominent EI measures used in the literature, as well as specifically in applied (e.g., health care) contexts. The emotional intelligence measures we included were those that measured both overall EI as well as more specific EI constructs common to multiple measures (e.g., those related to perceiving emotions in self and others, regulating emotions in self and others and strategically utilizing emotions). The second aim was to identify individual studies that have explored the validity and reliability of the specific emotional intelligence measures identified.

### Inclusion Criteria

Four main inclusion criteria were applied to select literature: (a) focus on adult samples, (b) use of reputable, peer-reviewed journal articles, (c) use of an EI scale, and (d) where possible, use of a professional sample (e.g., health care professionals) rather than primarily student samples. The literature search therefore focused on empirical, quantitative investigations published in peer-reviewed journals. The articles reviewed therefore were generally methodologically sound and enabled a thorough analysis of some aspect of reliability or validity. We only reviewed articles published after 1990. Additionally, only papers in English were reviewed.

### Sources

Papers were identified by conducting searches in the following electronic databases: PsycINFO, Medline, PubMED, CINAHL (Cumulative Index for Nursing and Allied Health Literature), EBSCO host and Google Scholar. Individual journals were also scanned such as The Journal of Nursing Measurement and Psychological Assessment.

### Search Terms

When searching for emotional intelligence scales and related literature, search terms included: trait emotional intelligence, ability emotional intelligence, emotional intelligence scales, mixed emotional intelligence and emotional intelligence measures. Some common EI facet titles (e.g., self-awareness, self-regulation/self-management, social awareness, and relationship management) were also entered as search terms however this revealed far less relevant literature than searches based on EI terms. To access studies using professionals we also used terms such as workplace, healthcare, and nursing, along with emotional intelligence.

When searching for literature on the identified scales, the name of the respective scale was included in the search term (such as TEIQue scale) and the authors' names, along with terms such as workplace, organization, health care, nurses, health care professionals, to identify specific studies with a professional employee sample that utilized the specific scale. The terms validity and reliability were also used. Additionally, a similar search was conducted on articles that had cited the original papers. This search was done conducted utilizing Google Scholar. Table 2 summarizes the result of the first part of the literature review. It provides an overview of major Emotional Intelligence assessment measures, in terms of when they were developed, who developed them, what form of EI they measure, theoretical basis, test length and details regarding cost.

Tables 3–8 summarize research on the validity and reliability of the 6 tests included in Table 2. In these tables we summarize the methodology used in major studies assessing reliability and validity as well as the results from these studies.

Collectively, these tables indicate that all 6 of the measures we reviewed have received some support for their reliability and validity. Measures with extensive research include the MSCEIT, SREIT, and TEIQue, and EQ-I and those with less total research are the STEU/STEM and ESCI. Existing research does not indicate that these latter measures are any less valid or reliable that the others; on the contrary they are promising measures but require further tests of reliability and validity. As noted previously, this table confirms that the tests with the strongest *current* evidence for construct and predictive validity are the self-report/trait EI measures (TEIQue, EQ-I, and SREIT). We note that although there is evidence for construct validity of the SREIT based on associations with theoretically related constructs (e.g., alexithymia, optimism; see Table 4), some have suggested the measure is problematic due to its use of self-report questions that primarily measure ability based constructs (see Petrides and Furnham, 2000).

## Conclusion

In this article we have reviewed six widely used measures of EI and made recommendations regarding their appropriate use. This article was written primarily for researchers and practitioners who are not currently experts on EI and therefore we also clarified the difference between ability EI, trait EI and mixed EI. Overall, we recommend that users should use single, complete tests where possible and choose measures of EI most suitable for their purpose (i.e., choose ability EI when maximal performance is important and trait EI when typical performance is important). We also point out that, across the majority of emotion-related outcomes, trait EI tends to be a stronger predictor and consequently we suggest that new users of EI consider using a trait-based measure before assessing alternatives. The exception is in employment contexts where tests utilizing 360 degree assessment (primarily mixed measures) can also be very useful.

## Author Contributions

All authors listed have made a substantial, direct and intellectual contribution to the work, and approved it for publication.

### Conflict of Interest Statement

The authors declare that the research was conducted in the absence of any commercial or financial relationships that could be construed as a potential conflict of interest.
